# Live‐cell imaging of octaarginine‐modified polymer dots via single particle tracking

**DOI:** 10.1111/cpr.12556

**Published:** 2019-02-01

**Authors:** Yao Luo, Yuping Han, Xingjie Hu, Min Yin, Changfeng Wu, Qian Li, Nan Chen, Yun Zhao

**Affiliations:** ^1^ College of Life Sciences Sichuan University Chengdu China; ^2^ Division of Physical Biology and Bioimaging Center, Shanghai Institute of Applied Physics Chinese Academy of Sciences Shanghai China; ^3^ Development and Regeneration Key Lab of Sichuan Province, Department of Anatomy and Histology and Embryology Chengdu Medical College Chengdu China; ^4^ School of Public Health Guangzhou Medical University Guangdong China; ^5^ Department of Chemistry Shanghai Normal University Shanghai China; ^6^ Department of Biomedical Engineering Southern University of Science and Technology Shenzhen China

**Keywords:** endosomal escape, live‐cell imaging, octaarginine peptide, polymer dots, single particle tracking

## Abstract

**Objectives:**

Nanocarriers can greatly enhance the cellular uptake of therapeutic agents to regulate cell proliferation and metabolism. Nevertheless, further application of nanocarriers is often limited by insufficient understanding of the mechanisms of their uptake and intracellular behaviour.

**Materials and methods:**

Fluorescent polymer dots (Pdots) are coated with synthetic octaarginine peptides (R8) and are analysed for cellular uptake and intracellular transportation in HeLa cervical cancer cells via single particle tracking.

**Results:**

Surface modification with the R8 peptide efficiently improves both cellular uptake and endosomal escape of Pdots. With single particle tracking, we capture the dynamic process of internalization and intracellular trafficking of R8‐Pdots, providing new insights into the mechanism of R8 in facilitating nanostructure‐based cellular delivery. Furthermore, our results reveal R8‐Pdots as a novel type of autophagy inducer.

**Conclusions:**

This study provides new insights into R8‐mediated cellular uptake and endosomal escape of nanocarriers. It potentiates biological applications of Pdots in targeted cell imaging, drug delivery and gene regulation.

## 1 INTRODUCTION

Nanotechnology has shown great potential in biomedical applications.^1^, ^2^, ^3^, ^4^ Numerous nanoparticles are developed and exploited as biosensors, diagnostic imaging probes or vehicles of various therapeutic reagents.^5^, ^6^, ^7^, ^8^, ^9^, ^10^ Fluorescent semiconducting polymer dots (Pdots) attract growing attention as ideal theranostic agents because of their good biocompatibility and outstanding optical properties, including high quantum yield and extraordinary photostability.^11^ Pdots have been broadly applied in cell labelling, super‐resolution cell imaging and single particle tracking.^12^, ^13^ More recently, near‐infrared fluorescent Pdots are investigated for long‐term tracking of engrafted MSCs in vivo.^14^ In addition to bioimaging, hydrophilic Pdots can form stable complexes with small interfering RNA (siRNA) and regulate gene expression in cancer cells.^15^ Insights into the intracellular behaviour and mechanism of nanoparticles are important for the design and improvement of nanocarriers and imaging probes for biomedical applications.^16^, ^17^, ^18^ Our recent work demonstrates that Pdots adopt distinct routes for endocytosis and intracellular trafficking in epithelial cells and macrophages. Although Pdots can be ingested in large amount by macrophages rapidly, the amount and speed of Pdots uptake by epithelial cells are much more limited. Moreover, following endocytosis, majority of Pdots are transported and destined into lysosomes, implying that bioactive cargos, such as DNA, RNA and proteins, are unlikely to keep their intracellular functionality.^19^

Many strategies have been developed to improve cellular uptake of nanoparticles and to avoid lysosomal degradation.^20^, ^21^ Coating with cationic lipids or attaching with specific targeting ligands can both increase the interaction with cell surface and enhance cellular uptake.^22^, ^23^ Another intensively studied strategy for endosomal escape of NPs is “proton sponge effect” based on cationic polymers that cause endosome osmotic swelling and disruption of the endosome membrane.^24^ However, these approaches are often deleterious to cells. Therefore, a simple and effective method to enhance the cellular uptake and to avoid lysosomal degradation of nanocarriers without producing cytotoxicity is highly required. Previous studies have used biomimetic cell‐penetrating peptides (CPPs) such as TAT, polylysine or polyarginine to deliver nanoparticles into living cells.^25^, ^26^ CPPs are often derived from viral proteins and possess the ability to cross cell membranes.^27^, ^28^ Nevertheless, further application of CPPs is limited by insufficient understanding of the mechanisms of their uptake and intracellular behaviour.^29^ Live‐cell imaging provides visible evidence of the trafficking and functionality of delivered therapeutics.^30^ In this study, we coat fluorescent Pdots with synthetic octaarginine peptides (R8) to analyse R8‐mediated cellular uptake and intracellular transportation in living HeLa human cervical cancer cells. Compared to unmodified Pdots that take hours to enter epithelial cells, significant amount of R8‐Pdots enter cells with minutes. Interestingly, R8 modification does not change the endocytic route of Pdots. Single particle tracking reveals that the process of R8‐Pdots internalization can be divided into several stages. Our results also show that R8‐Pdots avoid lysosomal localization with increased cytoplasmic distribution, which helps to retain the functionality of biomolecules. Moreover, we identify Pdots‐induced upregulation of autophagy in HeLa cells for the first time. Importantly, R8‐Pdots also increase autophagy levels in HeLa cells, implying that R8‐Pdots have potential to regulate cellular homeostasis directly in addition to function as imaging probes and carriers of therapeutic agents.

## 2 MATERIALS AND METHODS

### 2.1 Materials

Poly (styrene‐co‐maleic anhydride) (PSMA, Mn = 1700) and anhydrous tetrahydrofuran (THF, 99.9%) were purchased from Sigma‐Aldrich. Poly [(9,9‐dioctylfluorenyl‐2,7‐diyl)‐co‐(1,4‐benzo‐{2,1,3}‐thiadiazole)] (PFBT, MW = 10 000, polydispersity 1.7) was obtained from ADS Dyes (Quebec, Canada). Octaarginine peptides were purchased from Jie Li Bio. HeLa cell lines were purchased from Cell Bank of Chinese Academy of Sciences (Shanghai). Minimum essential media (MEM), Dulbecco's modified Eagle's medium (DMEM) and foetal bovine serum (FBS) were from Gibco, Invitrogen. Chlorpromazine (CPZ), methyl‐β‐cyclodextrin (mβCD) and EIPA were purchased from Sigma‐Aldrich (St. Louis, MO, USA). RFP‐LAMP1 plasmid was acquired from Addgene (plasmid # 1817).

### 2.2 Preparation and characterization of Pdots

Pdots were synthesized using a modified precipitation method. THF solution (5 mL) containing conjugated polymers (0.5 mg) and PSMA (0.2 mg) was quickly injected into 10 mL deionized water, and the mixture was subsequently sonicated for 2 minutes. THF was removed by partial vacuum evaporation. The resulting solution was concentrated by continuous heating, followed by filtration through a 0.22‐μm filter to remove fraction of aggregates.

For R8 modification, the as‐prepared Pdots were diluted to a concentration of 20 μg/mL before mixed with R8 at a molar ratio of 1:1000 in HEPES buffer before adding 2 × MEM media of the same volume.

The apparent hydrodynamic size and the zeta potential of nanoparticles were measured using a Zetasizer (nano ZS90, Malvern Instruments). For transmission electron microscopy, solution containing Pdots was dropped onto carbon‐coated copper grids to evaporate excess solvent and examined with TEM (Jeol 2010, 200 KV). Fluorescence spectra were obtained by an Edinburgh FS920 fluorescence spectrometer.

### 2.3 Cell culture and treatment

HeLa cells were grown in DMEM supplemented with 10% heat‐inactivated FBS and antibiotics (100 g/mL of streptomycin and 100 g/mL of penicillin) at 37°C with humidified atmosphere (5% CO_2_). Cells were seeded one day before Pdots incubation. The concentration was 5 μg/mL for R8‐Pdots and 20 μg/mL for unmodified Pdots. For inhibitor treatment, cells were pre‐treated for 1 hour with low temperature (4°C, 5 μg/mL CPZ, 10 μg/mL EIPA and 7 mM mβCD, respectively. For lysosome and autophagosome labelling, HeLa cells (1 × 10^6^) were transfected with 1 μg of the RFP‐LAMP1 or LC3‐RFP plasmids using lipofectamine 3000 (Invitrogen) according to the manufacturer's instructions.

### 2.4 Flow cytometry

Before measurements, cell media was removed and cells were washed for three times with PBS. Next, 0.2 mL trypsin (Invitrogen) was added to each sample and incubated for 1 minute at 37°C before 0.5 mL MEM was added. Cell suspensions were transferred into tubes before analysed using a FACS Calibur flow cytometer (Amnis ImageStream Mark II; MERCK MILLIPORE, Burlington, MA, USA). Consistent gating based on cell size and granularity (forward and side scatter) was applied to select the fluorescence signals of counted cells. At least 10 000 cells were counted for each sample, and experiments were performed in triplicates.

### 2.5 Western blotting

Cells were harvested using the SDS‐loading sample buffer and boiled. Protein samples were then analysed by 12% SDS‐polyacrylamide gel electrophoresis (PAGE) and blotted to PVDF membranes. The blots were blocked for 30 minutes using 5% non‐fat milk in phosphate‐buffered saline with 1% Tween 20 (PBST) buffer (0.1% Tween 20) and then incubated overnight at 4°C with the indicated primary antibodies anti‐LC3 (Novus Biologicals, Littleton, CO, USA). After washing three times with PBST, the blots were probed with a goat anti‐rabbit HRP secondary antibody. The membranes were developed using the chemiluminescent HRP substrate (Millipore) and visualized by the bioimaging system (Syngene G: Box).

### 2.6 Live‐cell imaging and single particle tracking

After incubation with R8‐Pdots for indicated time, images were acquired using a laser confocal microscope (Leica TCS SP8, Germany) equipped with a live‐cell incubator and collected with a HC × PL APO 63×, 1.4 NA oil‐immersion objective. R8‐Pdots was excited with a 488 nm Ar‐Kr laser, RFP was excited with a 561 nm helium‐neon laser, and Hoechst 33258‐labelled nuclei were excited with a 405 nm diode laser, respectively. The imaging channels were set at 500‐550, 570‐620 and 450‐500 nm, respectively. Three‐dimensional single particle tracking was performed using a DeltaVision Elite deconvolution scanning fluorescence microscope.^31^ Z‐stacks were collected at 0.2‐μm intervals over 2 μm. The images were deconvolved and superimposed.

### 2.7 Image analysis

Fluorescence images were analysed using ImageJ software (US National Institutes of Health). To quantify the colocalization ratio of two fluorescent signals, tMr values (the thresholded Mander's coefficients) indicating the percentage of green signals colocalized with red signals in merged images were calculated. Values represent mean ± SE based on analysis of randomly selected 20 cells. To measure the signal distribution inside cells, we divided the image into 9*9 squares and counted the average fluorescence intensity in each square using ImageJ scripts. For single particle tracking, the trajectories of red signals and green signals were built by pairing spots in each frame using single particle tracking plug‐in of ImageJ.

## 3 RESULTS

### 3.1 Preparation of R8‐modified Pdots

**Figure 1:**
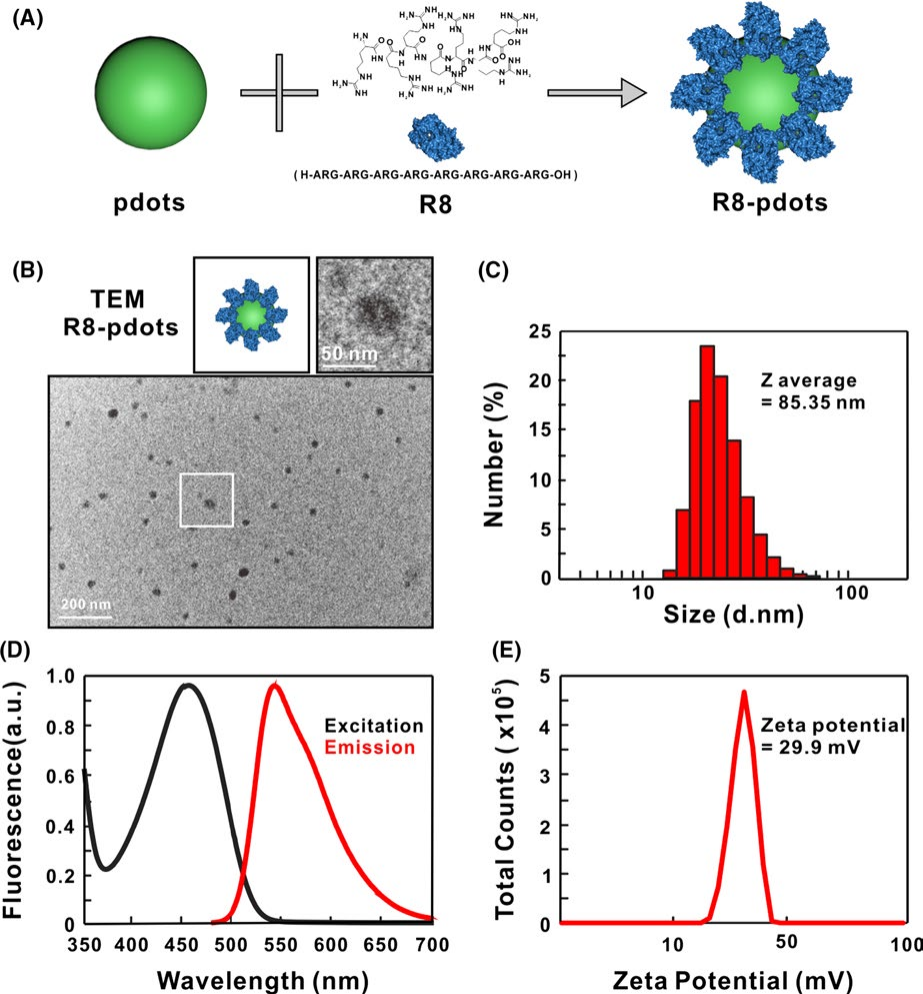
Preparation and characterization of R8‐Pdots. A, Schematic illustration of preparation of R8‐modified Pdots by adsorption. B, Representative TEM images of R8‐Pdots. C, Hydrodynamic diameter of Pdots was measured by dynamic light scattering. D, Excitation and emission spectra of R8‐Pdots. E, Zeta potential of R8‐Pdots was determined in water

Cell‐penetrating peptides (CPPs) are short peptides that can facilitate cellular uptake of a broad range of targets. R8 is a type of representative cationic CPPs and has been utilized to enhance the delivery of various nanocarriers, including liposomes, gold nanoparticles, quantum dots and near‐infrared Polymer dots.^32^, ^33^, ^34^ R8 is chosen for Pdots modification in this study. We have recently confirmed the biocompatibility of poly (9,9‐dioctylfluorenyl‐2,7‐diyl)‐co‐(1,4‐benzo‐{2,1′,3}‐thiadiazole) PFBT Pdots. PFBT‐conjugated Pdots are incubated with R8 peptides that can adsorb onto the surface through electrostatic interactions (Figure 1A). According to the results of transmission electron microscopy (TEM), most particles possess diameters in the range of 25 ± 10 nm and are uniformly spherical (Figure 1B). Dynamic light scattering (DLS) analysis shows the average hydrodynamic diameter is 85 nm, which is similar to unmodified Pdots (Figure 1C). Also, R8 modification does not change the fluorescent spectrum of Pdots: an adsorption peak around 460 nm and a maximum fluorescence emission around 540 nm are detected (Figure 1D). Significantly, Figure 1E shows R8‐Pdots have a positive zeta potential of +29 mV, indicating that negatively charged Pdots that have a zeta potential of −35 mV (Figure S1) are successfully modified by R8 through electrostatic adsorption.^35^

### 3.2 Cellular uptake of Pdots is enhanced by R8 modification

**Figure 2:**
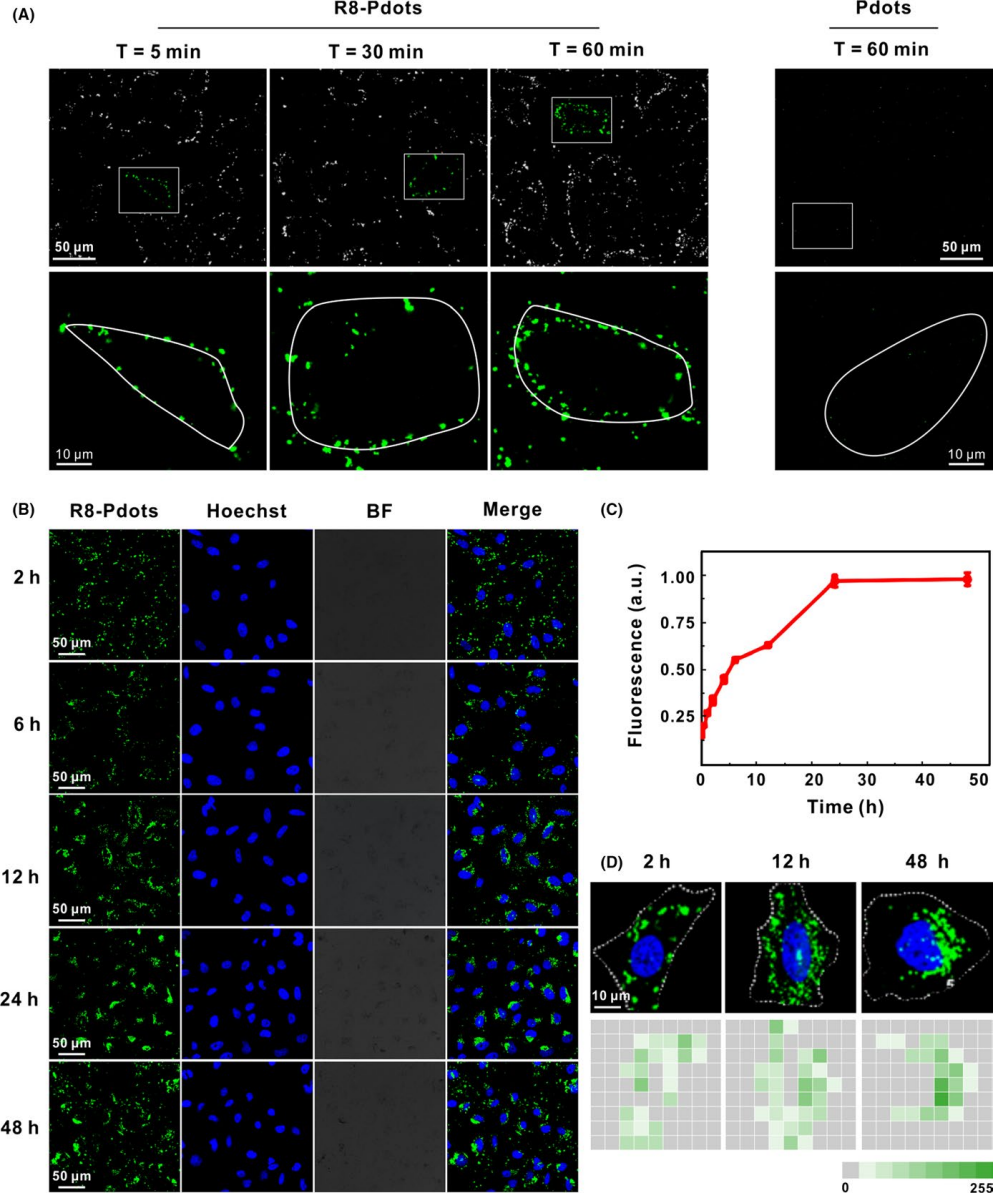
Time‐dependent live‐cell imaging of R8‐Pdots. A, HeLa cells were incubated with 5 μg/mL R8‐Pdots (left) or 20 μg/mL unmodified Pdots for 60 minutes and observed using confocal microscopy. Lower panel shows magnified images of the square region in the upper panel. Cellular periphery is outlined according to bright‐field imaging. B, HeLa cells were incubated with 5 μg/mL R8‐Pdots for up to 48 hours. Intracellular fluorescence was observed using confocal microscopy at indicated time points. C, Fluorescence of internalized R8‐Pdots was quantified by flow cytometry analysis. D, Representative images of ingested R8‐Pdots at 2, 12 and 48 hours were divided into 9*9 squares, and the average fluorescence intensity in each square was counted using ImageJ scripts

We firstly inspect whether R8‐Pdots can efficiently enter HeLa cells. According to our previous observation, Pdots can be ingested rapidly by macrophages, whereasHeLa cells have a relatively slow intake process. Fluorescent signals are hardly detectable in HeLa cells in the first 6 hours of incubation.^19^ Strikingly, cellular uptake of R8‐Pdots by HeLa cells happens within minutes. Fluorescent signal appears on the cell membrane within 5 minutes, and intracellular signals increase quickly over time. At 60 minutes, bright fluorescent signals can be observed inside the cell (Figure 2A). In contrast, cells incubated with unmodified Pdots show neglectable fluorescence, indicating that coating with R8 peptides significantly accelerated the rate of cellular uptake of Pdots. We further monitor the dynamics of R8‐Pdots uptake of for 48 hours using both confocal fluorescent microscope and flow cytometer. The fluorescent intensity increases with time until it reaches the maximum at 24 hours and remains stable (Figure 2B,C). These results confirm that R8 modification enhances both the amount and speed of Pdots internalization by HeLa cells. Notably, the intracellular distribution of fluorescent signals shows different pattern at the early and late stage following cellular uptake. At 2 hours, majority of the fluorescent puncta located close to the cell membrane, while they concentrate around the nucleus at 48 hours (Figure 4D). These data indicate R8‐Pdots are transported gradually from the cell membrane to the perinuclear region, most likely along the microtubule, which is consistent with our previous observation that transportation of endocytic vesicles depends on intact microtubules.^36^

### 3.3 Single particle tracking of endocytic R8‐Pdots

**Figure 3:**
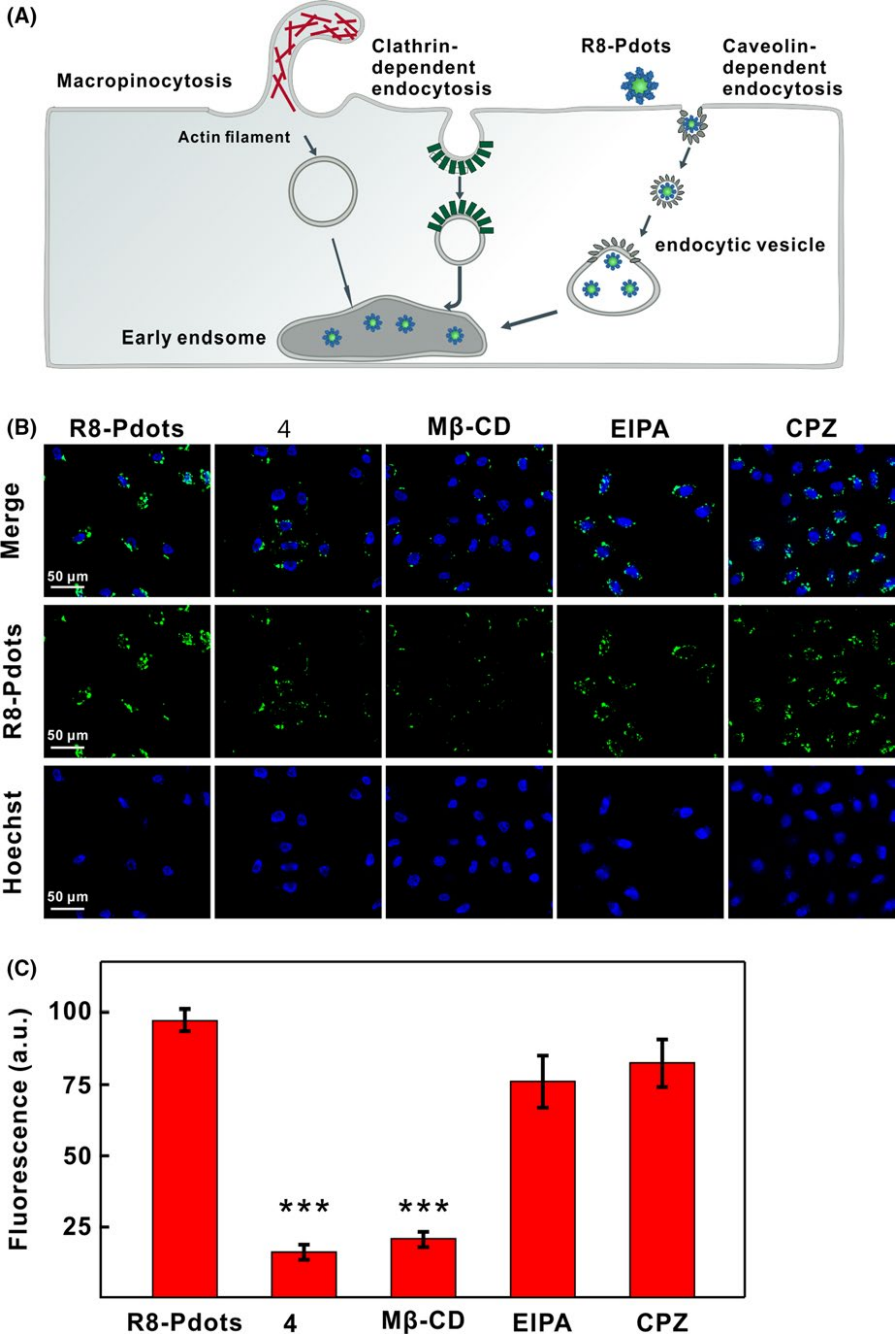
Endocytic pathway of R8‐Pdots. A, Schematic illustration of various endocytotic routes and internalization of R8‐Pdots by HeLa cells. B, Confocal images of HeLa cells treated with indicated pharmacological inhibitors. Cells were pre‐treated with low temperature (4°C), mβ‐CD, EIPA or CPZ for 30 minutes, followed by incubation with 5 μg/mL R8‐Pdots for another 60 minutes. C, Intracellular fluorescent signals of cells treated in the presence of different inhibitors were quantified using the ImageJ software (20 cells analysed)

Since R8‐Pdots show much faster uptake rate than their unmodified counterpart, we set out to determine the endocytic mechanisms of R8‐Pdots. Currently, the mechanism of CPP‐facilitated endocytosis is controversially discussed and poorly understood.^25^, ^37^ Nanoparticles can enter cells through multiple endocytic pathways, including phagocytosis, macropinocytosis, clathrin‐dependent endocytosis, caveolin‐dependent endocytosis, clathrin‐ and caveolin‐independent pathways (Figure 3A).^38^ We examine R8‐Pdots uptake by HeLa cells in the presence of selective inhibitors of distinct endocytic routes. No significant intracellular fluorescence is undetectable when HeLa cells are incubated with R8‐Pdots at 4°C, indicating that R8‐Pdots are internalized through energy‐dependent endocytosis (Figure 3B). Methyl‐β‐cyclodextrin (mβCD) could specifically inhibit caveolin‐dependent endocytosis by depleting cholesterol and disrupting the structures of lipid raft on the cell membrane. 5‐(N‐Ethyl‐N‐isopropyl)‐amiloride (EIPA) is an inhibitor of macropinocytosis. Chlorpromazine (CPZ) is commonly utilized to block clathrin‐dependent endocytosis.^39^ Compared to untreated cells, pre‐treatment of HeLa cells with EIPA or CPZ causes no obvious reduction of intracellular fluorescence intensity, whereas mβCD treatment results in 80% reduction of internalized R8‐Pdots (Figure 3B,C). These results are confirmed by the quantitative analysis of flow cytometer, where mβCD treatment causes strong reduction of R8‐Pdots uptake (Figure S2). Taken together, these results indicate that although R8 modification accelerates and enhances the uptake of Pdots, it does not change the endocytosis pathway.

**Figure 4:**
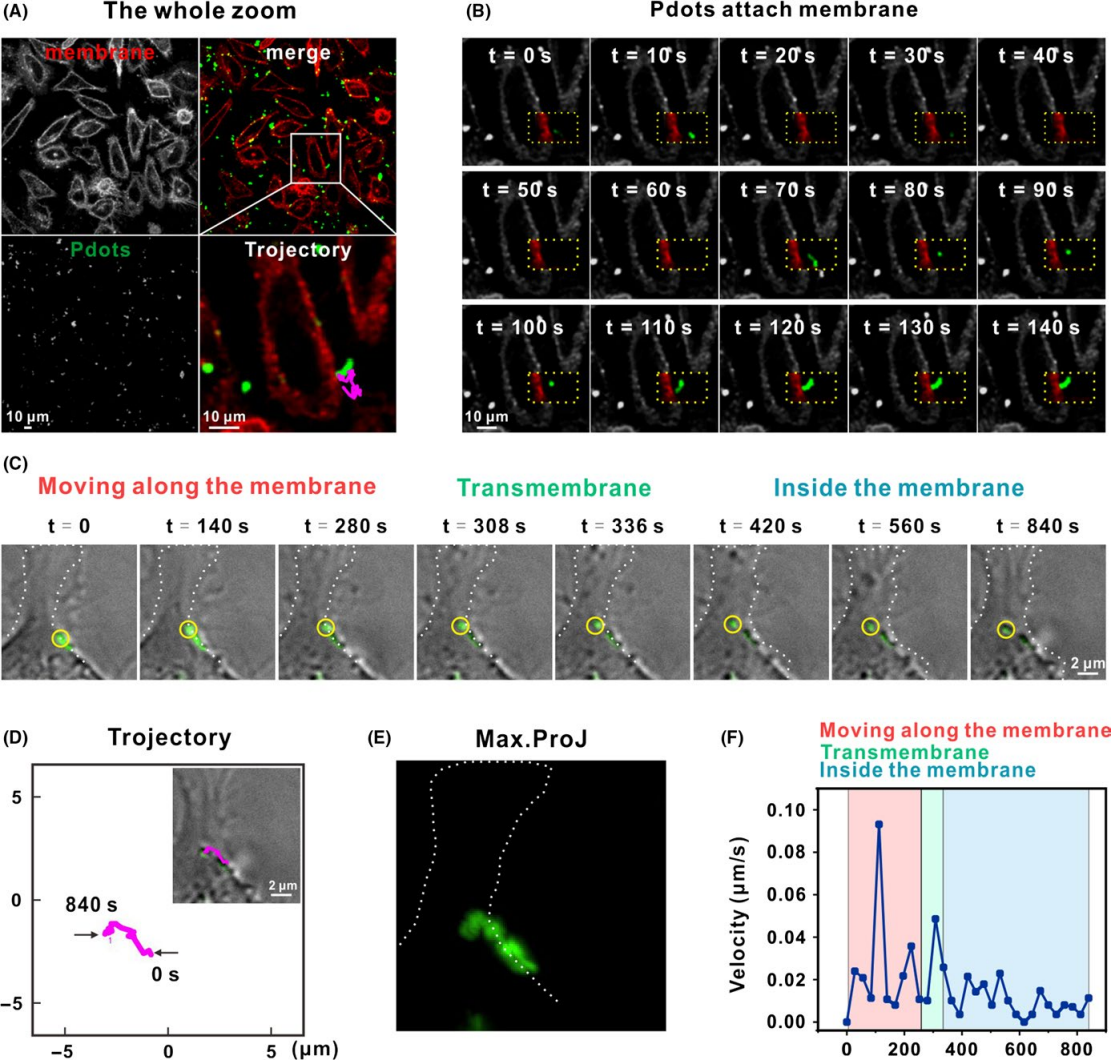
Single particle tracking of dynamic R8‐Pdots internalization. A, Confocal images of R8‐Pdots (green) internalization. Cell membrane was stained with Dil (red) 5 minutes. The samples were observed within 1 hour after staining. Trajectory of a representative particle was shown with enlarged view (one frame per 2 seconds, with a total of 140 seconds; also see Video S1). Trajectories at different time points were shown in (B). C, The whole internalization process of a R8‐Pdot particle (indicated with a yellow circle) was imaged using a DeltaVision deconvolution scanning fluorescence microscope. Z‐stacks were collected at 0.2‐μm intervals over 2 μm (one frame per 28 seconds, with a total of 840 seconds; also see Video S2). The whole process was divided into three stages. Cellular periphery is outlined according to bright‐field imaging. D, Trajectory of the representative particle. Max trajectory and instantaneous speed of selected R8‐Pdots particle were shown in (E) and (F), respectively

To have a detailed look of the whole internalization process of R8‐Pdots, we perform real‐time imaging to trace the dynamic movement of R8‐Pdots at single particle level. Cell membrane is labelled with a hydrophilic fluorescent dye 1, 1′‐dioctadecyl‐3,3,3′,3′‐tetramethylindocarbocyanine perchlorate (Dil) to monitor the interaction with Pdots. Time‐lapse movie is acquired using confocal microscope (Figure 4A,B; Video S1, Δ*t* = 2 seconds, total time = 140 seconds). It shows clearly the whole process of a representative R8‐Pdots particle moving towards the cell and attaching to the membrane. We also record the dynamic process of internalization of R8‐Pdots via time‐lapse three‐dimensional live‐cell imaging. The whole process can be divided into three stages: R8‐Pdots moves on the edge of cell membrane before crosses the membrane, and moves in the cytoplasm (Figure 4C; Video S2, Δ*t* = 28 seconds, total time = 840 seconds). Trajectories of the representative particle (indicated by a yellow circle) are shown (Figure 4D,E). Before crossing the cell membrane, the R8‐Pdots particle moves with a velocity between 0 and 0.1 μm/s (Figure 4F). After the particle comes into contact with the cell membrane, its moving velocity slowed down significantly. The retention of R8‐Pdots particle on the cell membrane lasts for more than 300 seconds before it moves into the cytoplasm, suggesting that R8‐Pdots interact with specific proteins on cell surface before internalization (Figure 4F).

### 3.4 Endosomal escape of R8‐modified Pdots

**Figure 5:**
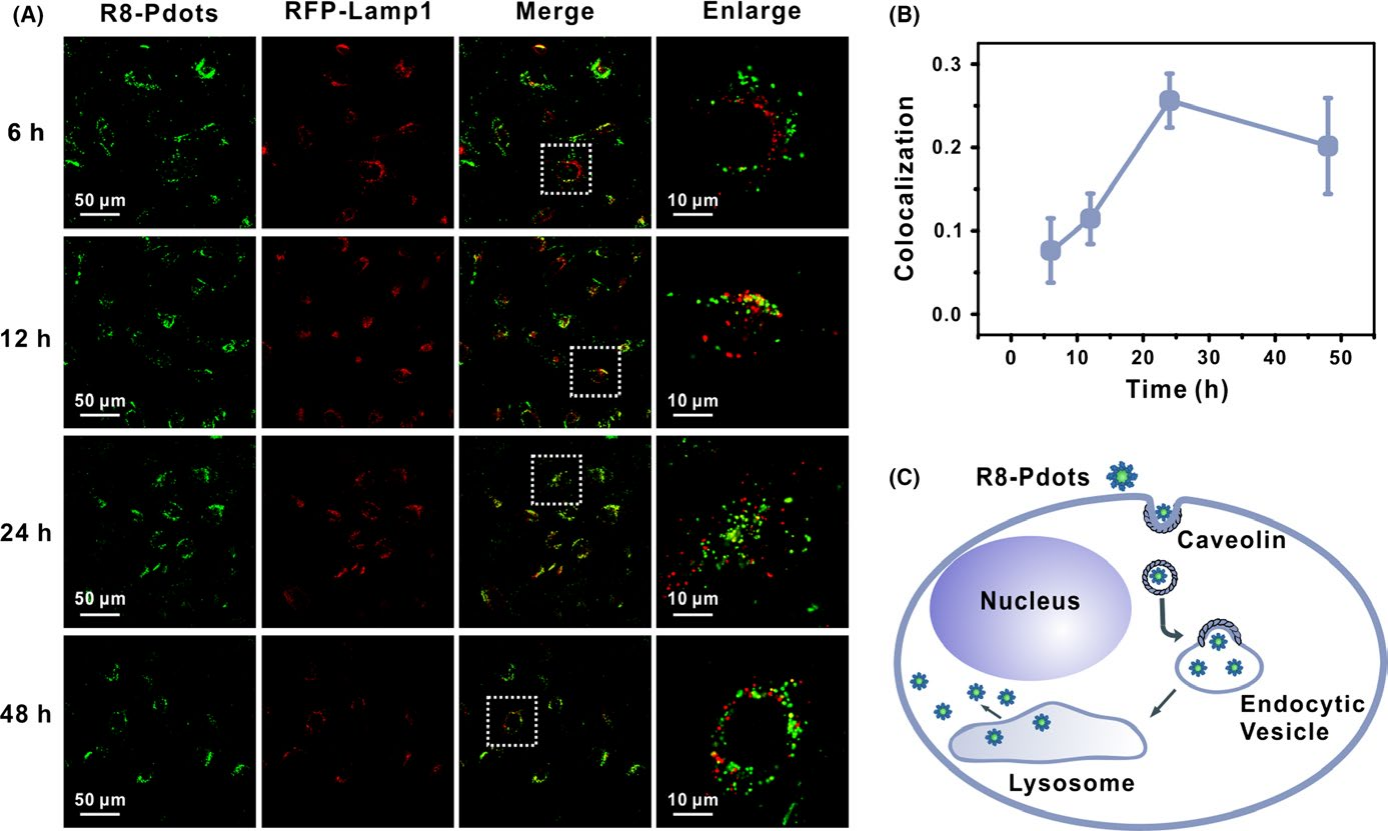
R8 modification facilitates endosomal escape of Pdots. HeLa cells expressing RFP‐Lamp1 (red) were incubated with 5 μg/mL R8‐Pdots (green) and imaged by confocal microscope at indicated time points. Right panel shows magnified images of the square region in the left panel. B, Colocalization ratio of R8‐Pdots with LAMP1 was quantified using the ImageJ software (20 cells analysed). C, Schematic illustration of intracellular distribution and transportation of R8‐Pdots

Having elucidated the endocytic pathway and process of R8‐Pdots, we set out to investigate their intracellular behaviour. Unmodified Pdots enter endo‐lysosomal pathway and end up in lysosomes, which greatly restricts their potential in delivery of bioactive cargos like nucleic acids and proteins. Multiple CPPs, including R8 peptides, have been previously reported to facilitate escape of conjugated cargos from lysosomes.^40^ We examined the colocalization between R8‐Pdots and acidic endo‐lysosomal organelles in HeLa cells. Lysosomes are labelled by expression of red fluorescent protein (RFP)‐fused marker protein lysosomal‐associated membrane protein 1 (LAMP‐1). After transfection of the LAMP1‐RFP plasmid for 24 hours, HeLa cells are incubated with R8‐Pdots for 6, 12, 24 and 48 hours, respectively, and examined using a confocal microscope. Colocalization between R8‐Pdots and LAMP1‐RFP is less than 10% after 6‐hour incubation and the ratio reaches the maximum value of 25% after 24 hours (Figure 5A,B). In comparison, more than 50% of unmodified Pdots colocalize with LAMP1‐RFP in 6 hours and the ratio increase to 80% with prolonged incubation (Figures S3 and S4). These data demonstrate clearly that R8 peptides efficiently change the intracellular fate of Pdots in HeLa cells by preventing their trafficking into lysosomes (Figure 5C). Notably, R8‐modified liposomes also exhibit increased endosomal escape and enhanced expression levels of encapsulated DNA plasmids.^41^ However, different endocytic mechanisms have been reported for R8‐liposomes, including clathrin‐mediated endocytosis and macropinocytosis. CPP‐facilitated endocytosis and intracellular trafficking processes are complicated and can be regulated by multiple aspects including types of CPPs, density and conformation of CPPs on the surface of nanoparticles, and targeting cell types.^37^ Our results suggest that modification with high‐density R8 peptides via electrostatic adsorption can accelerate Pdots uptake in HeLa cells without changing the endocytic route, presumably by promoting their interaction with certain membrane receptors. Furthermore, following caveolin‐dependent endocytosis, endocytic vesicles containing R8‐Pdots are not destined to lysosomes. This property will greatly benefit Pdots‐based delivery of bioactive cargos into cytosol and keep their functionality.

### 3.5 R8‐Pdots induces autophagy

**Figure 6:**
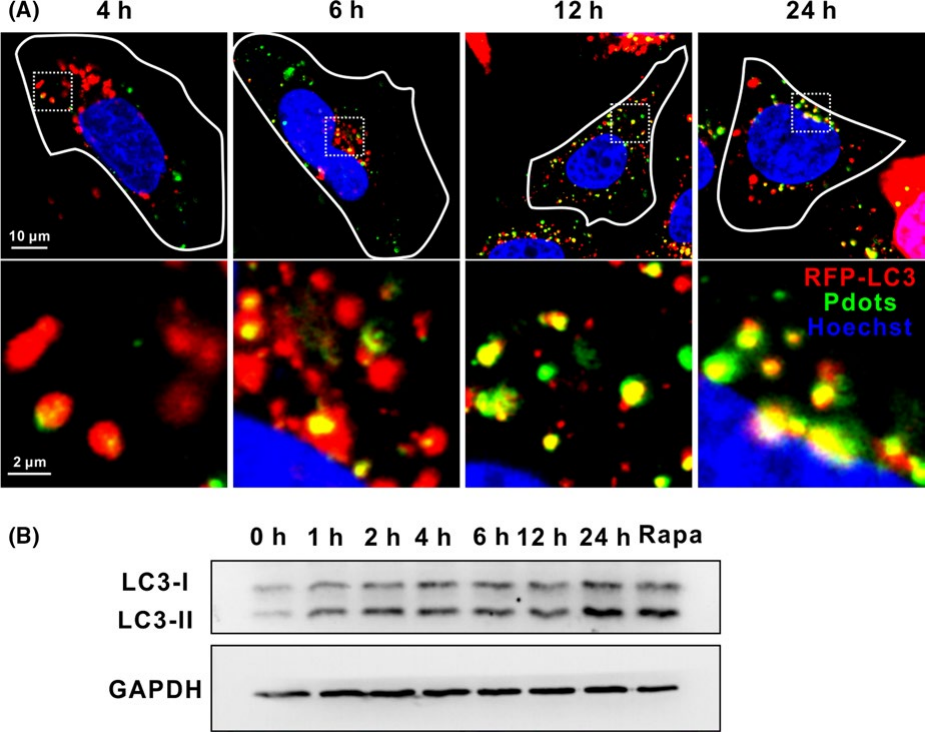
R8‐Pdots induce autophagy. HeLa cells expressing RFP‐LC3 (red) were incubated with 20 μg/mL Pdots (green) and imaged by confocal microscope at indicated time points. Lower panel shows magnified images of the square region in the upper panel. B, HeLa cells were incubated with 5 μg/mL R8‐Pdots for indicated time. Cells treated with 100 μM rapamycin were included as a positive control. Protein levels of LC3 were analysed by Western blotting. GAPDH was included as loading control

R8‐modification appears to be a simple but effective strategy to enhance Pdots‐mediated delivery and maintain the functionality of bioactive cargos in epithelial cells. Nevertheless, it remains unknown whether Pdots themselves interfere with the intracellular membrane trafficking system and affect the homeostatic status. The endocytosis and intracellular trafficking of other nanoparticles have been reported to regulate various cellular pathways including Wnt and TGF‐β pathways, hypoxia and autophagy.^42^, ^43^, ^44^ Particularly, autophagy plays critical pathophysiological roles and is a common cellular stress response.^45^, ^46^, ^47^ The whole autophagic flux is closely related to the formation, transportation and fusion of intracellular membrane bound structures. Therefore, it is likely to be regulated by fluctuations of the trafficking process. Several nanomaterials have been reported to elicit autophagy.^48^, ^49^ We examine the autophagic phenotype in Pdots‐treated HeLa cells. During the formation of autophagosomes, microtubule‐associated protein light chain 3 (LC3) is converted from a cytoplasmic form (LC3‐I) to a phosphatidylethanolamine (PE)‐conjugated form (LC3‐II), which is then localized onto the outer membrane of autophagosomes. Hence, the conversion and the punctuated distribution of LC3‐II protein are commonly used as a marker for autophagy.^50^, ^51^ HeLa cells ectopically expressing LC3‐RFP are incubated with 20 μg/mL Pdots and examined by confocal microscope at various time points. Increased number of punctuated LC3‐RFP is observed after 4 hours. Colocalization analysis reveals that more Pdots are targeted to autophagosomes with prolonged incubation time (Figure 6A). Immunoblotting assay indicates gradually accumulation of LC3‐II within 24 hours (Figure S5A). Importantly, R8‐Pdots incubation at lower concentration (5 μg/mL) also significantly increases LC3‐II levels. Furthermore, the amount of ingested Pdots is directly related with upregulation of autophagy since R8‐Pdots‐induced LC3‐II appears after only 1‐hour incubation (Figure 6B). Since R8‐Pdots are not destined for lysosomal degradation, they may not directly enter the double‐membraned vesicles for autophagy induction. We hypothesize that R8‐Pdots‐induced autophagy is the result of active endocytosis and accompanying ROS stress. Further study is needed to illustrate the mechanism and physiological relevance of Pdots‐induced autophagy. Nanoparticles‐induced autophagy has been exploited for synergetic treatment of tumours and neurodegenerative diseases.^52^, ^53^ Therefore, our results identify R8‐Pdots as novel autophagy inducer for the first time, which opens new routes to design Pdots‐based theranostic reagents.

## 4 DISCUSSION

We report the use of R8‐conjugated Pdots to investigate the complicated behaviour of nanoparticles in live cells, which is important for developing advanced cellular imaging probes and nanocarriers. Single particle tracking indicates that R8 modification promotes immediate binding of Pdots to negatively charged cell membranes and triggers caveolin‐dependent endocytosis, with increased amount and accelerated speed. Following endocytosis, the internalized R8‐Pdots are actively transported towards the perinuclear region. We also find that majority of R8‐Pdots avoid to be destined to the lysosomes. The enhanced cellular uptake, as well as effective endosomal escape of R8‐Pdots, makes them an ideal candidate for the delivery of bioactive cargos into epithelial cells. Furthermore, we identify Pdots as a novel autophagy inducer in HeLa cells and R8 modification does not compromise this activity. To our knowledge, this is the first report of autophagy induced by Pdots. Considering the critical role of autophagy in maintaining cellular homeostasis and its connection with various diseases, our results shed new light on the development of Pdots‐based nanoreagents that combine cellular imaging, targeted delivery and manipulation of cellular metabolism.

## CONFLICT OF INTEREST

The authors declare no conflict of interest.

## References

[1] Li J , Song S , Liu X , et al. Enzyme‐based multi‐component optical nanoprobes for sequence‐specific detection of DNA hybridization. Adv Mater. 2008;20(3):497‐500.

[2] He Y , Zhong Y , Su Y , et al. Water‐dispersed near‐infrared‐emitting quantum dots of ultrasmall sizes for in vitro and in vivo imaging. Angew Chem Int Ed Engl. 2011;50(25):5695‐5698. 21557405 10.1002/anie.201004398

[3] Lin M , Wang J , Zhou G , et al. Programmable engineering of a biosensing interface with tetrahedral DNA nanostructures for ultrasensitive DNA detection. Angew Chem Int Ed Engl. 2015;54(7):2151‐2155. 25556850 10.1002/anie.201410720

[4] Ma W , Xie X , Shao X , et al. Tetrahedral DNA nanostructures facilitate neural stem cell migration via activating RHOA/ROCK2 signalling pathway. Cell Prolif. 2018;e12503. 30091500 10.1111/cpr.12503 PMC6528883

[5] Kim D , Shin K , Kwon SG , Hyeon T . Synthesis and biomedical applications of multifunctional nanoparticles. Adv Mater. 2018;e1802309. 30133009 10.1002/adma.201802309

[6] Wang Z , Fu Y , Kang Z , et al. Organelle‐specific triggered release of immunostimulatory oligonucleotides from intrinsically coordinated DNA‐metal‐organic frameworks with soluble exoskeleton. J Am Chem Soc. 2017;139(44):15784‐15791. 29024595 10.1021/jacs.7b07895

[7] Pei H , Liang L , Yao G , Li J , Huang Q , Fan C . Reconfigurable three‐dimensional DNA nanostructures for the construction of intracellular logic sensors. Angew Chem Int Ed Engl. 2012;51(36):9020‐9024. 22887892 10.1002/anie.201202356

[8] Zhong R , Tang Q , Wang S , et al. Self‐assembly of enzyme‐like nanofibrous G‐molecular hydrogel for printed flexible electrochemical sensors. Adv Mater. 2018;30(12):e1706887. 29388269 10.1002/adma.201706887

[9] Chen L , Chao J , Qu X , et al. Probing cellular molecules with PolyA‐based engineered aptamer nanobeacon. ACS Appl Mater Interfaces. 2017;9(9):8014‐8020. 28221021 10.1021/acsami.6b16764

[10] Lai W , Ren L , Tang Q , et al. Programming chemical reaction networks using intramolecular conformational motions of DNA. ACS Nano. 2018;12(7):7093‐7099. 29906089 10.1021/acsnano.8b02864

[11] Chen H , Fang X , Jin Y , et al. Semiconducting polymer nanocavities: porogenic synthesis, tunable host‐guest interactions, and enhanced drug/siRNA delivery. Small. 2018;14(21):e1800239. 29682859 10.1002/smll.201800239

[12] Yu J , Wu C , Sahu SP , Fernando LP , Szymanski C , McNeill J . Nanoscale 3D tracking with conjugated polymer nanoparticles. J Am Chem Soc. 2009;131(51):18410‐18414. 20028148 10.1021/ja907228q

[13] Wu C , Chiu DT . Highly fluorescent semiconducting polymer dots for biology and medicine. Angew Chem Int Ed Engl. 2013;52(11):3086‐3109. 23307291 10.1002/anie.201205133 PMC5616106

[14] Chen D , Li Q , Meng Z , et al. Bright polymer dots tracking stem cell engraftment and migration to injured mouse liver. Theranostics. 2017;7(7):1820‐1834. 28638470 10.7150/thno.18614 PMC5479271

[15] Liu Y , Gunda V , Zhu X , et al. Theranostic near‐infrared fluorescent nanoplatform for imaging and systemic siRNA delivery to metastatic anaplastic thyroid cancer. Proc Natl Acad Sci USA. 2016;113(28):7750‐7755. 27342857 10.1073/pnas.1605841113 PMC4948349

[16] Chen N , Wei M , Sun Y , et al. Self‐assembly of poly‐adenine‐tailed CpG oligonucleotide‐gold nanoparticle nanoconjugates with immunostimulatory activity. Small. 2014;10(2):368‐375. 23963797 10.1002/smll.201300903

[17] Gao Z , Deng S , Li J , et al. Sub‐diffraction‐limit cell imaging using a super‐resolution microscope with simplified pulse synchronization. Sci Chi Chem. 2017;60(10):1305‐1309.

[18] Su Y , Peng T , Xing F , Li D , Fan C . Nanoplasmonic biological sensing and imaging. Acta Chim Sinica. 2017;75(11):1036‐1046.

[19] Han Y , Li X , Chen H , et al. Real‐time imaging of endocytosis and intracellular trafficking of semiconducting polymer dots. ACS Appl Mater Interfaces. 2017;9(25):21200‐21208. 28586196 10.1021/acsami.7b05662

[20] Varkouhi AK , Scholte M , Storm G , Haisma HJ . Endosomal escape pathways for delivery of biologicals. J Control Release. 2011;151(3):220‐228. 21078351 10.1016/j.jconrel.2010.11.004

[21] Sahay G , Querbes W , Alabi C , et al. Efficiency of siRNA delivery by lipid nanoparticles is limited by endocytic recycling. Nat Biotechnol. 2013;31(7):653‐658. 23792629 10.1038/nbt.2614 PMC3814166

[22] Canton I , Battaglia G . Endocytosis at the nanoscale. Chem Soc Rev. 2012;41(7):2718‐2739. 22389111 10.1039/c2cs15309b

[23] Semple SC , Akinc A , Chen J , et al. Rational design of cationic lipids for siRNA delivery. Nat Biotechnol. 2010;28(2):172‐176. 20081866 10.1038/nbt.1602

[24] Vermeulen L , Brans T , Samal SK , et al. Endosomal size and membrane leakiness influence proton sponge‐based rupture of endosomal vesicles. ACS Nano. 2018;12(3):2332‐2345. 29505236 10.1021/acsnano.7b07583

[25] Lee JH , Zhang A , You SS , Lieber CM . Spontaneous internalization of cell penetrating peptide‐modified nanowires into primary neurons. Nano Lett. 2016;16(2):1509‐1513. 26745653 10.1021/acs.nanolett.6b00020

[26] Folini M , Bandiera R , Millo E , et al. Photochemically enhanced delivery of a cell‐penetrating peptide nucleic acid conjugate targeting human telomerase reverse transcriptase: effects on telomere status and proliferative potential of human prostate cancer cells. Cell Prolif. 2007;40(6):905‐920. 18021178 10.1111/j.1365-2184.2007.00470.x PMC6760699

[27] Copolovici DM , Langel K , Eriste E , Langel U . Cell‐penetrating peptides: design, synthesis, and applications. ACS Nano. 2014;8(3):1972‐1994. 24559246 10.1021/nn4057269

[28] Bernkop‐Schnurch A . Strategies to overcome the polycation dilemma in drug delivery. Adv Drug Deliv Rev. 2018; 10.1016/j.addr.2018.07.017. 30059702

[29] Ruan G , Agrawal A , Marcus AI , Nie S . Imaging and tracking of tat peptide‐conjugated quantum dots in living cells: new insights into nanoparticle uptake, intracellular transport, and vesicle shedding. J Am Chem Soc. 2007;129(47):14759‐14766. 17983227 10.1021/ja074936k

[30] Seitz G , Warmann SW , Fuchs J , et al. Imaging of cell trafficking and metastases of paediatric rhabdomyosarcoma. Cell Prolif. 2008;41(2):365‐374. 18336479 10.1111/j.1365-2184.2008.00520.x PMC6495802

[31] Qu X , Zhu D , Yao G , et al. An exonuclease III‐powered, on‐particle stochastic DNA walker. Angew Chem. 2017;56(7):1855‐1858. 28079956 10.1002/anie.201611777

[32] Khalil IA , Kogure K , Futaki S , Harashima H . Octaarginine‐modified liposomes: enhanced cellular uptake and controlled intracellular trafficking. Int J Pharm. 2008;354(1–2):39‐48. 18242018 10.1016/j.ijpharm.2007.12.003

[33] Nakase I , Akita H , Kogure K , et al. Efficient intracellular delivery of nucleic acid pharmaceuticals using cell‐penetrating peptides. Acc Chem Res. 2012;45(7):1132‐1139. 22208383 10.1021/ar200256e

[34] Meng Z , Guo L , Li Q . Peptide‐coated semiconductor polymer dots for stem cells labeling and tracking. Chemistry. 2017;23(28):6836‐6844. 28370830 10.1002/chem.201700002

[35] Shao X‐R , Wei X‐Q , Song X , et al. Independent effect of polymeric nanoparticle zeta potential/surface charge, on their cytotoxicity and affinity to cells. Cell Prolif. 2015;48(4):465‐474. 26017818 10.1111/cpr.12192 PMC6496505

[36] Wang T , Wang L , Li X , et al. Size‐dependent regulation of intracellular trafficking of polystyrene nanoparticle‐based drug‐delivery systems. ACS Appl Mater Interfaces. 2017;9(22):18619‐18625. 28497682 10.1021/acsami.7b05383

[37] Foerg C , Ziegler U , Fernandez‐Carneado J , et al. Decoding the entry of two novel cell‐penetrating peptides in HeLa cells: lipid raft‐mediated endocytosis and endosomal escape. Biochemistry. 2005;44(1):72‐81. 15628847 10.1021/bi048330+

[38] Oh N , Park JH . Endocytosis and exocytosis of nanoparticles in mammalian cells. Int J Nanomedicine. 2014;9(suppl 1):51‐63. 24872703 10.2147/IJN.S26592 PMC4024976

[39] Liu M , Li Q , Liang L , et al. Real‐time visualization of clustering and intracellular transport of gold nanoparticles by correlative imaging. Nat Commun. 2017;8:15646. 28561031 10.1038/ncomms15646 PMC5460036

[40] Toriyabe N , Hayashi Y , Harashima H . The transfection activity of R8‐modified nanoparticles and siRNA condensation using pH sensitive stearylated‐octahistidine. Biomaterials. 2013;34(4):1337‐1343. 23141897 10.1016/j.biomaterials.2012.10.043

[41] Song L , Liang X , Yang S , et al. Novel polyethyleneimine‐R8‐heparin nanogel for high‐efficiency gene delivery in vitro and in vivo. Drug Deliv. 2018;25(1):122‐131. 29265887 10.1080/10717544.2017.1417512 PMC6058572

[42] Yi H , Wang Z , Li X , et al. Silica nanoparticles target a Wnt signal transducer for degradation and impair embryonic development in zebrafish. Theranostics. 2016;6(11):1810‐1820. 27570552 10.7150/thno.16127 PMC4997238

[43] Li X , Song L , Hu X , et al. Inhibition of epithelial‐mesenchymal transition and tissue regeneration by waterborne titanium dioxide nanoparticles. ACS Appl Mater Interfaces. 2018;10(4):3449‐3458. 29318884 10.1021/acsami.7b18986

[44] Wang L , Wang Z , Li X , et al. Deciphering active biocompatibility of iron oxide nanoparticles from their intrinsic antagonism. Nano Res. 2018;11(5):2746‐2755.

[45] Popp L , Segatori L . Differential autophagic responses to nano‐sized materials. Curr Opin Biotechnol. 2015;36:129‐136. 26340102 10.1016/j.copbio.2015.08.016

[46] Deng Y , Zhu L , Cai H , Wang G , Liu B . Autophagic compound database: a resource connecting autophagy‐modulating compounds, their potential targets and relevant diseases. Cell Prolif. 2018;51(3):e12403. 29094410 10.1111/cpr.12403 PMC6528906

[47] Xie T , Li SJ , Guo MR , et al. Untangling knots between autophagic targets and candidate drugs, in cancer therapy. Cell Prolif. 2015;48(2):119‐139. 25650136 10.1111/cpr.12167 PMC6496907

[48] Stern ST , Adiseshaiah PP , Crist RM . Autophagy and lysosomal dysfunction as emerging mechanisms of nanomaterial toxicity. Part Fibre Toxicol. 2012;9:20. 22697169 10.1186/1743-8977-9-20 PMC3441384

[49] Song W , Soo Lee S , Savini M , Popp L , Colvin VL , Segatori L . Ceria nanoparticles stabilized by organic surface coatings activate the lysosome‐autophagy system and enhance autophagic clearance. ACS Nano. 2014;8(10):10328‐10342. 25315655 10.1021/nn505073u

[50] Li X , Chen N , Su Y , et al. Autophagy‐sensitized cytotoxicity of quantum dots in PC12 cells. Adv Healthc Mater. 2014;3(3):354‐359. 24039192 10.1002/adhm.201300294

[51] Wang Z , Luo Y , Han Y , Chen N , Fan C . Nanoparticle‐based regulation and imaging of cell autophagy. Sci Chi Chem. 2017;47(6):778‐786.

[52] Peynshaert K , Manshian BB , Joris F , et al. Exploiting intrinsic nanoparticle toxicity: the pros and cons of nanoparticle‐induced autophagy in biomedical research. Chem Rev. 2014;114(15):7581‐7609. 24927160 10.1021/cr400372p

[53] Wang L , Li X , Han Y , et al. Quantum dots protect against MPP+‐induced neurotoxicity in a cell model of Parkinson’s disease through autophagy induction. Sci Chi Chem. 2016;59(11):1486‐1491.

