# Supplementary material for: Live‐cell imaging of octaarginine‐modified polymer dots via single particle tracking
Source: Cell Prolif. 2019 Feb 1;52(2):e12556. doi: 10.1111/cpr.12556 (PMC6496536; doi:10.1111/cpr.12556)
Supplement: Supplementary file 1 [file CPR-52-e12556-s001.docx]

**Supplementary Information**

**Live-Cell Imaging of Octaarginine Modified Polymer Dots via Single Particle Tracking**

**Running title:** Imaging and single particle tracking of R8-Pdots

*Yao Luo^1,2^, Yuping Han^2,3^, Xingjie Hu^2,4^, Min Yin^2,5^, Changfeng Wu^6^, Qian Li^2,*^, Nan Chen^2,5,*^, Yun Zhao^1,*^*

^1^College of Life Sciences, Sichuan University, Chengdu 610064, China

^2^Division of Physical Biology and Bioimaging Center, Shanghai Institute of Applied Physics, Chinese Academy of Sciences, Shanghai 201800, China

^3^ Development and Regeneration Key Lab of Sichuan Province, Department of Anatomy and Histology and Embryology, Chengdu Medical College, Chengdu 610500, China

^4^School of Public Health, Guangzhou Medical University, Guangdong 511436, China

^5^Department of Chemistry, Shanghai Normal University, Shanghai 200234, China

^6^Department of Biomedical Engineering, Southern University of Science and Technology, Shenzhen, Guangdong 518055, China

Y. Luo and Y. Han should be considered as joint first author.

* Correspondence: [zhaoyun@scu.edu.cn](mailto:zhaoyun@scu.edu.cn); [chennan@sinap.ac.cn](mailto:chennan@sinap.ac.cn); [liqian@sinap.ac.cn](mailto:liqian@sinap.ac.cn)

Figures S1−S5

Video S1 (AVI)

Video S2 (AVI)


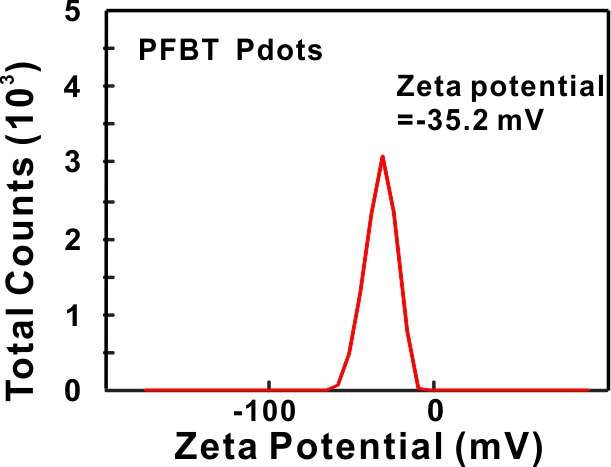


Figure S1. Zeta potential of unmodified PFBT Pdots was determined in water.


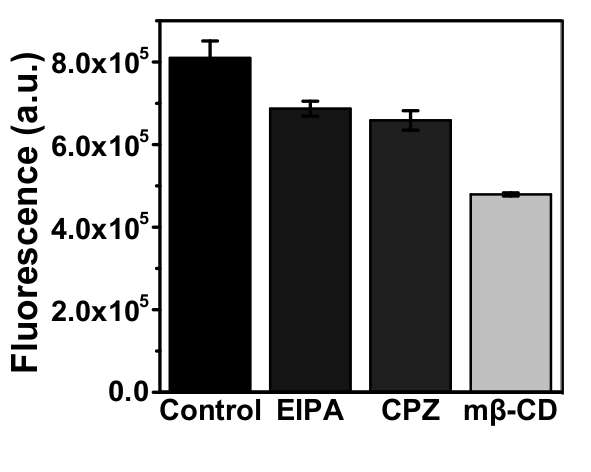


Figure S2. HeLa cells were pretreated with mβ-CD, EIPA or CPZ for 30 minutes, followed by incubation with 5 μg/mL R8-Pdots for another 4 hours. Fluorescence of internalized R8-Pdots was quantified by flow cytometry analysis.


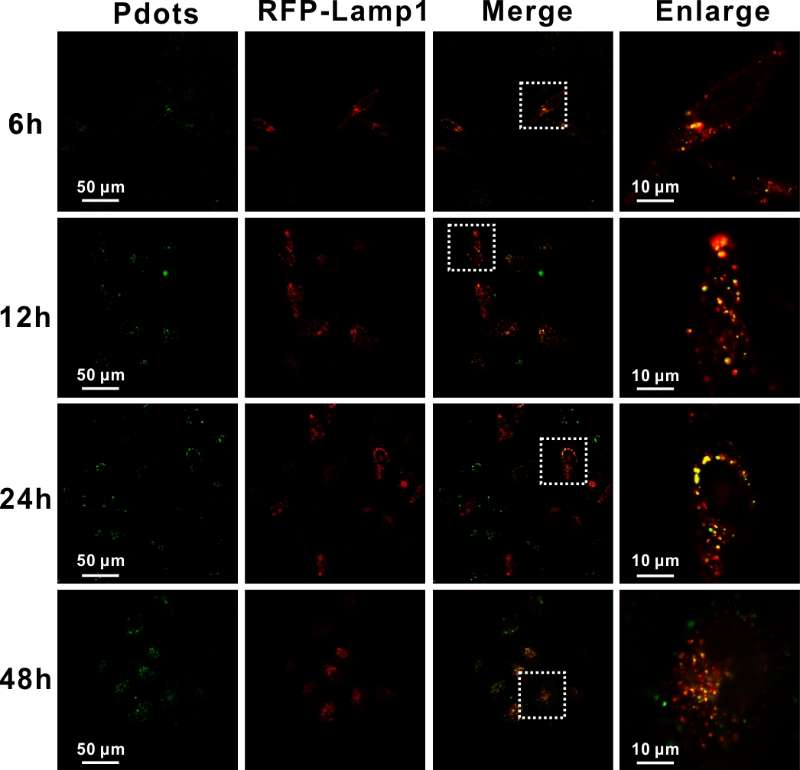


Figure S3. Unmodified Pdots were transported into lysosomes. HeLa cells expressing RFP-Lamp1 (red) were incubated with 20 μg/mL Pdots (green) and imaged by confocal microscope at indicated time points. Right panel show magnified images of the square region in the left panel.


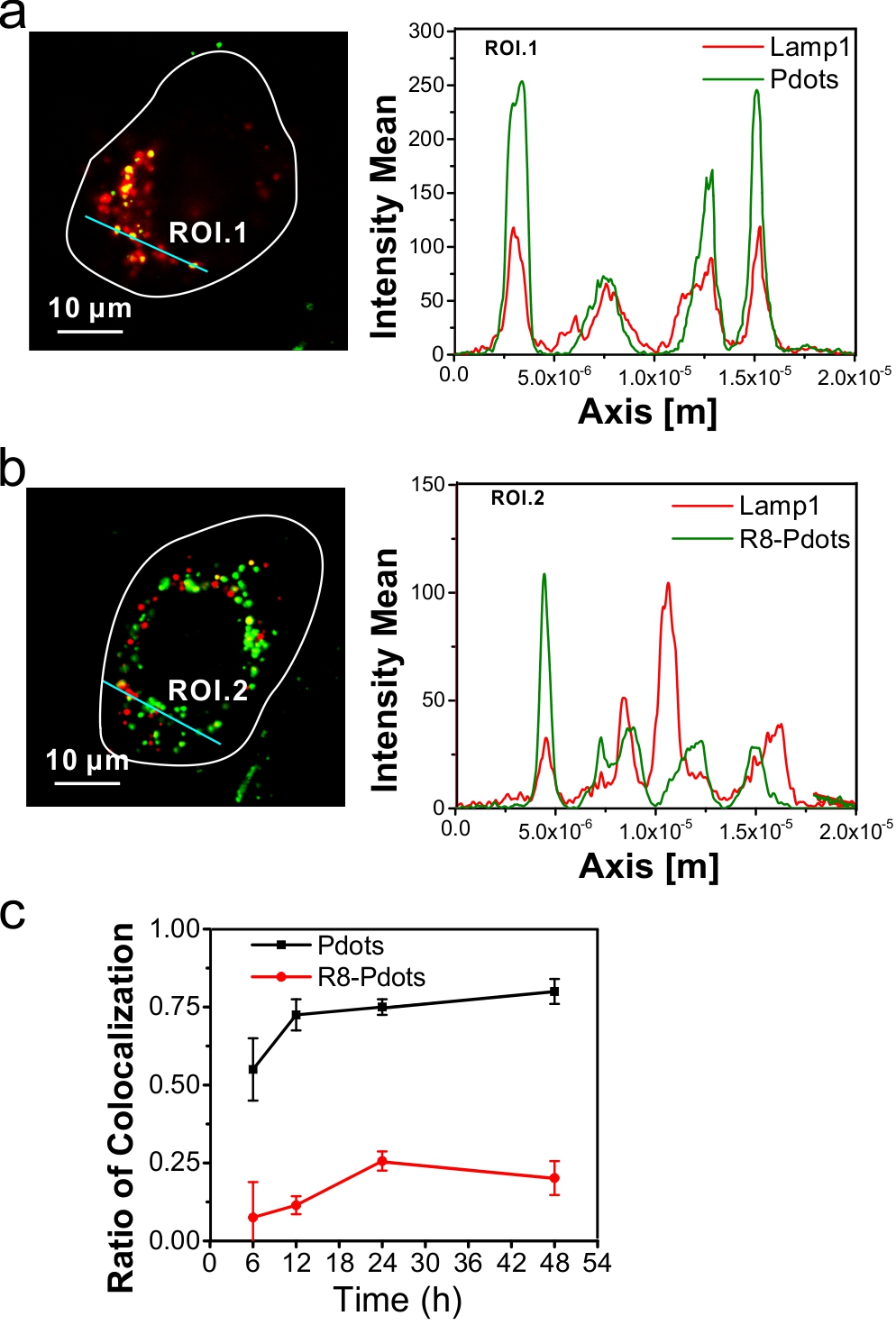


Figure S4. Intracellular distribution of Pdots and R8-Pdots. (a) The colocalization between LAMP1 and Pdots was examined by confocal microscopy and line profiling of fluorescence intensities of RFP-LAMP1 (red) and Pdots or R8-Pdots (green). (c) Colocalization ratio of LAMP1 with Pdots (black) or R8-Pdots (red) quantified using the ImageJ software (20 cells analyzed).


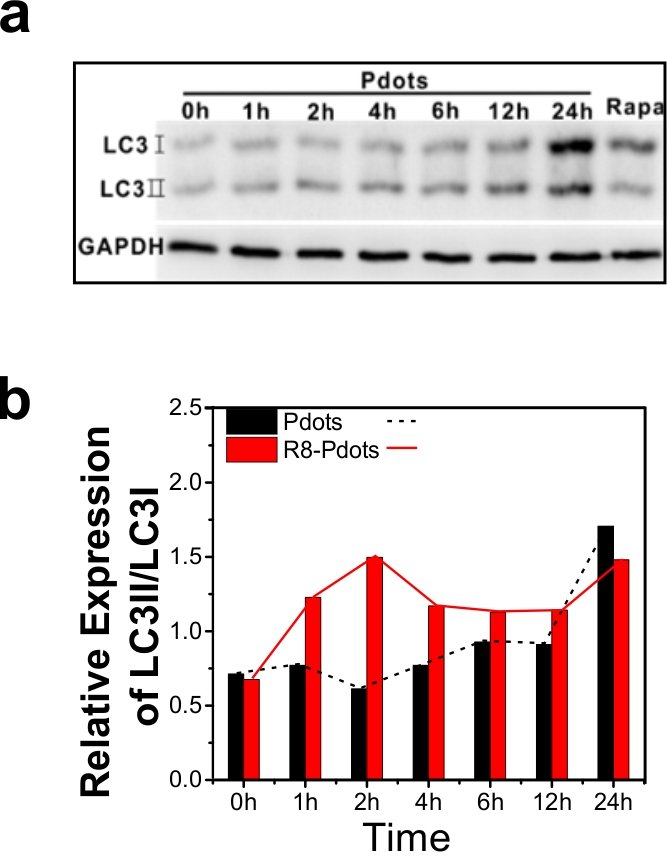


Figure S5. R8 modification accelerates Pdots-induced autophagy. (a) HeLa cells were incuabed with 20 μg/mL Pdots for indicated time. Protein levels of LC3 were analyzed by western blotting. (b) Both Pdots and R8-Pdots-induced conversions between LC3-I and LC3-II were analyzed by immunoblotting and quantified using imageJ.
